# Metabolomic analysis reveals trimethylamine N-oxide as a biomarker for poor outcome of severe spontaneous intracerebral hemorrhage patients receiving surgical treatment

**DOI:** 10.3389/fneur.2025.1551239

**Published:** 2025-04-14

**Authors:** Xinguo Sun, Kaige Zheng, Shanjun Wang, Yunzhao Chen, Hongen Liu, Xindong Gu, Zitong Wu, Hui Lu, Shuo Wang, Qingyuan Liu, Zengguang Wang

**Affiliations:** ^1^Department of Neurosurgery, Tianjin Medical University General Hospital, Tianjin, China; ^2^Department of Neurosurgery, Binzhou People's Hospital, Binzhou, Shandong, China; ^3^Department of Neurosurgery, Beijing Tiantan Hospital, Capital Medical University, Beijing, China; ^4^China National Clinical Research Center for Neurological Diseases, Beijing, China; ^5^Department of Neurosurgery, Yidu Central Hospital of Weifang, Qingzhou, Shandong, China; ^6^Department of Neurosurgery, Inner Mongolia Autonomous Region People's Hospital, Hohhot, Inner Mongolia, China

**Keywords:** spontaneous intracerebral hemorrhage, surgical treatment, poor outcome, metabolomic analysis, biomarker

## Abstract

**Background and objectives:**

Patients suffering from severe spontaneous intracerebral hemorrhage (SSICH) are at high risk of cardiocerebrovascular diseases postoperatively, which hugely affect patients’ long-term outcomes. Metabolic features could reflect the pathological change of the cardiocerebrovascular system and might serve as biomarkers for evaluating the risk of poor outcomes in SSICH patients. The current study aimed to find the early-warning biomarkers for poor outcomes in SSICH patients after surgery.

**Methods:**

Severe spontaneous intracerebral hemorrhage patients receiving surgical treatment from a referring hospital were prospectively included and formed the primary cohort after propensity score matching. The primary outcome is poor 180 days after hemorrhage (modified Rankin scale ≥4). Metabolomics analysis on 3-, 7-, and 30-day serum and cerebrospinal fluid samples after surgery revealed the dysregulated metabolites of SSICH patients within the primary cohort. Within the validation cohort of SSICH patients receiving surgical treatment from a multicenter, prospective cohort, dysregulated metabolites were validated and evaluated to see whether they could serve as biomarkers for 180-day poor outcomes by area under the curve (AUC).

**Results:**

The primary cohort included 20 SSICH patients with good 180-day outcome and 20 with poor outcome. Untargeted metabolomics analysis found 25 co-dysregulated metabolites, including trimethylamine N-oxide (TMAO), among 3-day, 7-day, and 30-day metabolism features between SSICH patients with poor outcome and good outcome after surgery. A good correlation was found in TMAO between serum and cerebrospinal fluid on 3rd day after surgery. Based on the validation cohort of 794 SSICH patients (147 patients had 180-day poor outcome), the targeted metabolomics analysis revealed increasing TMAO on 3rd day after surgery as a risk factor of poor outcomes (odds ratio, 4.7; 95%CI, 3.6–6.2; *p* < 0.001), with a good predictive value (AUC, 0.81).

**Conclusion:**

This study demonstrated increasing serum TMAO level as an early-warning biomarker for 180-day poor outcomes of SSICH patients receiving surgical treatment.

**Clinical trial registration:**

Chinese Clinical Trial Registry, ChiCTR1900024406, https://www.chictr.org.cn/showproj.html?proj=40640.

## Introduction

Severe spontaneous intracerebral hemorrhage (SSICH), characterized by extensive hematoma and escalating intracranial pressure, stands as the most perilous and fatal form of hemorrhagic stroke (1, 2). Timely surgical intervention has been validated to be effective for SSICH patients (3). However, some patients may have poor postoperative outcomes, and clinical deterioration is often observed as a late manifestation of brain injury (4, 5). Therefore, the early detection of biomarkers for adverse outcomes is of paramount importance. It enables healthcare professionals to strategically allocate appropriate resources and implement interventions, thereby enhancing the overall prognosis for postoperative SSICH patients.

Metabolomics could reflect the dynamic, multi-parameter metabolic responses of whole organisms to pathophysiological stimuli, which could reveal the biomarkers of brain injury after surgery in SSICH patients (6, 7). Prior studies found the relationship between abnormal metabolism and poor outcomes in patients with intracerebral hemorrhage and ischemic stroke (8–11). However, these studies were usually limited by single-center cross-sectional designs and small sample size and did not take patients receiving surgical treatment into consideration. Therefore, the metabolic characteristics of SSICH patients after surgery remained extensively unknown. A deep study of the metabolic characteristics of SSICH patients might reveal the biomarkers for a poor postoperative outcome.

This study aimed to discover early-warning biomarkers capable of forecasting the poor outcome of SSICH patients receiving surgical treatment. In the current study, we prospectively collected serum and cerebrospinal fluid (CSF) samples from SSICH patients receiving surgical treatment in a referring hospital and investigated the dynamic metabolic characteristics of SSICH patients postoperatively. We subsequently investigated the biomarkers of poor outcomes in SSICH patients postoperatively and validated their value based on a prospective, multi-center Chinese cohort.

## Materials and methods

This study was conducted in accordance with the STROBE protocol and approved by the institutional review board. All patients (or guardians) provided written informed consents.

Severe spontaneous intracerebral hemorrhage patients receiving surgical treatment were prospectively included from a referring hospital as the primary cohort. The inclusion criteria were listed as follows: (1) age of 18–75 years old; (2) severe intracerebral hemorrhage, which was diagnosed by radiological findings [supratentorial hematoma>30 mL, infratentorial hematoma >10 mL, midline shift >1 cm, or severe intraventricular hemorrhage [Graeb score >8] (12)]; (3) receiving surgical treatment, referring to craniotomy and minimally invasive surgery (endoscopic surgery and minimally invasive evacuation). This study further excluded patients with (1) cerebrovascular diseases such as intracranial aneurysm or cerebrovascular malformation; (2) intracranial tumors associated with hemorrhage; (3) hemorrhagic transformation of cerebral infarction; (4) intracerebral hemorrhage precipitated by venous thrombosis; (5) severe coagulation disorders such as hemophilia, or coagulation dysfunction due to malignant tumors and hypohepatia; (6) prior antithrombotic therapy (vitamin K antagonist and others) before hemorrhage; (7) death before hospital arrival or within a short period (6 h) post-admission; and (8) absence of follow-up data.

Severe spontaneous intracerebral hemorrhage patients from the SAP-ICH cohort (unique identifier: ChiCTR1900024406) were included as the validation cohort. The inclusive and exclusive criteria were the same as the primary cohort.

### Follow-up and outcome

All included SSICH patients were monitored through outpatient visits and telephonic consultations every 3 months until either death or 180 days post-hemorrhage. During each follow-up, investigators assessed the neurological functional state using the modified Rankin Score (mRS). The primary outcome was the neurological function outcome of SSICH patients on the 180th day after hemorrhage, evaluated using the mRS. A 180-day mRS of 4–6 was defined as a poor outcome, while the mRS of 0–3 was deemed as a good outcome. To avoid evaluation bias, in-hospital and follow-up records were collected and independently assessed by two experienced neurosurgeons with more than 10 years of work experience and blinded to patients’ information. All disagreements were resolved through consultation with a senior neurosurgeon (with more than 20 years of work experience).

### Propensity score matching of primary cohort

This study formed the primary cohort with minimal effects of confounding factors potentially related to postoperative outcome using the propensity score matching (PSM) to investigate the metabolic features of SSICH patients receiving surgical treatment. The propensity score was calculated by using logistic regression model with the following parameters: a target as 180-day poor outcome; the ratio of a good outcome to poor outcome = 1:1; nearest-neighbor matching method without replacement; caliper radius equal to a standard deviation of 0.1; and covariate balance to a standardized mean difference <0.1. The matching factors included age, male gender, diabetes mellitus, dyslipidemia, history of ischemic cerebrovascular and cardiovascular disease (ICCD), renal dysfunction, history of dual antiplatelet therapy (DAPT), Glasgow coma scale at admission, hematoma location and volume, IVH, and surgical methods (5, 13, 14). After PSM, a multivariate logistic model was then employed to validate no effect of the above confounding factors on 180-day poor outcomes.

### Sample collection

Patient blood samples were collected on the 3rd, 7th, and 30th days after surgery, and cerebrospinal fluid samples were collected on the 3rd day post-surgery. The decision to collect samples was based on a fixed time point based on clinical routine practice. After excluding blood samples with hemolysis, the remainder was centrifuged at 3,000 rpm for 10 min within 3 h after collection. All unused samples were quickly frozen in liquid nitrogen for 15 min and then transferred to a −80°C storage environment.

### Clinical data collection

The following baseline information was collected, including age, gender, comorbidities (including the history of dyslipidemia, diabetes mellitus, intracerebral hemorrhage, ischemic cerebrovascular or cardiovascular diseases and renal dysfunction), antiplatelet therapy before hemorrhage, tobacco and alcohol consumption, and laboratory examination after admission (platelet count, activated partial thromboplastin time [APTT], international normalized ratio [INR] and fibrinogen), GCS at admission, as well as surgical methods.

Ischemic cerebrovascular and cardiovascular diseases include transient ischemic attacks, ischemic stroke, coronary artery disease, and myocardial infarction. Renal dysfunction covered prior acute and chronic renal failure, and renal dysfunction post-admission [glomerular filtration rate <90 mL/(min × 1.73 m^2^)] (15). Antiplatelet therapy before hemorrhage was recognized if the patient received antiplatelet therapy for over 7 days but discontinued such therapy less than 7 days before hemorrhage. All included patients were categorized as none, single antiplatelet therapy, and dual antiplatelet therapy (DAPT). The surgical method was classified as craniotomy, craniotomy + craniectomy, endoscopic evacuation, and minimally invasive evacuation.

Alcohol use was classified as regular (one or more drinks per week) and non-regular (less than one drink per week, or no drink). Smoking status was defined as current if ongoing or smoking cessation less than 1 year, otherwise as non-current (smoking cessation more than 1 year, or no smoking).

Thrombocytopenia was defined as platelet count <100 × 10^9^/L (16). Coagulation disorder was defined as APTT ≥60 s, or INR ≥ 1.5, or fibrinogen <1.5 mg/dL.

Our previous clinical-radiological decision tree model was used to evaluate the risk of 180-day poor outcomes (output data as the probability of 180-day poor outcomes) of all SSICH patients in the validation cohort (5).

### Radiological images analysis

Radiological assessments were executed based on CT source images. A neurosurgeon and a neuroradiologist, both with work experience exceeding 5 years and blinded to patients’ information, independently evaluated radiological features. These features included hematoma location, hematoma volume, and intraventricular hemorrhage (IVH). Hematoma location was stratified into supratentorial and infratentorial categories. Hematoma volume was calculated employing the 3DSlicer (a free software, available at https://www.slicer.org). The average of hematoma volumes measured by the two investigators was utilized for further analysis.

### Management of SSICH patients

Upon admission, all SSICH patients received standard care in accordance with the guidelines, including blood pressure management, airway management, dynamic CT monitoring, among others (1). Surgical treatment decisions were made based on the consensus between physicians and patients’ guardians. In this study, surgical interventions were carried out by senior neurosurgeons (with work experience exceeding 10 years) immediately after admission or within 24 h after the indications for surgery arose. For patients receiving surgical treatment, CT scans were routinely performed at the 4th, 24th, and 48th hours after surgery. Furthermore, if the state of consciousness deteriorated, an immediate CT scan would be conducted.

### Untargeted metabolomics analysis

HPLC-grade methyl tert-butyl ether (MTBE) and methanol (MeOH) and n-hexane were purchased from Merck (Darmstadt, Germany). MilliQ water (Millipore, Bradford, United States) was used in all experiments. Sodium chloride and phosphate were bought from Sigma-Aldrich (St. Louis, MO, United States). Methanol solution of 15% boron trifluoride were bought from RHAWN (Shanghai, China). All of the standards were purchased from Sigma-Aldrich (St. Louis, MO, United States). The stock solutions of standards were prepared at the concentration of 1 mg/mL in MTBE. All stock solutions were stored at −20°C and diluted with MTBE to working solutions before analysis.

After the sample was thawed, an amount of 0.05 mL of the sample was mixed with 150 μL MeOH, 200 μL MTBE, and 50 μL 36% phosphoric acid/water (precooled at −20°C). The sample was vortexed for 3 min under the condition of 2,500 r/min, and centrifuged at 12000 r/min for 5 min at 4°C. Two hundred microliter supernatant was put into a new centrifuge tube to blow dry and 300 μL methanol solution of 15% boron trifluoride was added to vortex for 3 min under the condition of 2,500 r/min, and then it was kept in an oven at 60°C for 30 min. Later, it was cooled to room temperature, and then 500 μL n-hexane and 200 μL saturated sodium chloride solution was accurately added. After vortexing for 3 min and centrifugation at 4°C and 12,000 r/min for 5 min, 100 μL n-hexane layer solution was transferred for further GC–MS analysis.

The sample deviants were analyzed using a GC-EI-MS system (GC, Agilent 8,890 and MS, 5977B System). The analytical conditions were as follows, GC: column, DB-5MS capillary column (30 m × 0.25 mm × 0.25 μm, Agilent); Carrier gas, high purity helium (purity >99.999%); The heating procedure was started at 40°C (2 min), 30°C/min increased to 200°C (1 min), 10°C/min increased to 240°C (1 min), 5°C/min increased to 285°C (3 min); traffic: 1.0 mL/min; inlet temperature: 230°C; injection volume: 1.0 μL. Metabolites were detected by MetWare^1^ based on the Agilent 8,890-5977B GC–MS platform. Possible structures were inferred through database search including HMDB database, Massbank database, and METLIN database with exact molecular weights. Moreover, the retention time and fragmentation characteristic ions were obtained, and the metabolites’ structure was finally confirmed by using comparative analysis through progenesis QI software (WatersMilford, MA, United States).

When it comes to metabonomic analysis, the metabolites with a |log2(FC)| > 1 and *p* < 0.05 between the two groups were significant. The Analysis Module in MetaboAnalyst^2^ was used for metabolite classification and pathway enrichment analysis.

### Targeted metabolomics analysis

Multiple reaction monitoring was employed for targeted metabolomics analysis. To prepare targeted metabolomics of plasma samples, 20 μL plasma was aliquoted to a 1.5 mL Axygen tube and mixed with 80uL 10uM 13C-oleic acid in methanol. Then the supernatant was collected following a centrifugation at 20,000 g and 4°C for 10 min, which was analyzed after being injected onto a Kinetex c18 column (2.6 μm 50*2.1 mm. 00b-4462-AN Phenomenex, Torrance, CA) at a flow rate of 0.6 mL/min using an LC-20 AD Shimadazu pump system and a SIL-20AXR autosampler interfaced with an API 6500Q-TRAP mass spectrometer (AB SCIEX, Framingham, MA). A discontinuous gradient was generated to separate the analytes by mixing Solvent A (5 mM ammonium acetate in water) and Solvent B (acetonitrile) at different ratios. It started from 30% B and linearly increased to 90% B over 6.5 min, and then rose to 98% B in 0.1 min and remained for 2.5 min, and finally returned to 30% B. Analytes were monitored using electrospray ionization in the negative-ion mode through the multiple-reaction monitoring of precursors. The injection volume was 1 μL. The column temperature was maintained at 35°C, the optimized ionization parameters were: probe temperature 500°C, curtain gas 30 psi, nebulizing gas and heating gas 50 psi and ion spray voltage −4,500 kV. To get the precise concentration, a standard curve was performed. 20 μL standards with various concentrations (0–100 μM) were processed through the same procedure, and standard curves were acceptable when the coefficient of determination reached 0.99.

5-Hydroxyeicosatetraenoic acid (5-HETE), Trimethylamine N-oxide (TMAO), trimethylamine (TMA) and choline in serum and CSF were detected by using the multiple reaction monitoring based on the standard samples (Sigma-Aldrich, United States). Other metabolites in serum were detected by using multiple reaction monitoring based on the signal intensity.

### Statistical analysis

Statistical evaluations were conducted with SPSS software (version 24.0). Normally distributed continuous variables were represented as means and standard deviation, and medians (m) and inter-quartile range (IQR) for non-normally distributed variables. Categorical variables were shown as numbers (no.) and percentages (%). Differences between continuous variables were compared using Student’s *t*-tests or Wilcoxon rank sum tests, and differences in categorical variables by using chi-square tests or Fisher’s exact tests. Univariate and multivariate logistic regression analyses were performed to identify the factors related to the poor outcome of SSICH patients after surgical treatment. The result was presented as an odds ratio (OR) and 95% CI. The value of features or parameters to predict the risk of a 180-day poor outcome was evaluated by the receiver operate curve (ROC) and area under curve (AUC). To investigate whether metabolic markers could provide additional value to the previous model, we incorporated the risk of poor outcome assessed by the clinical-radiological model and metabolites for 180-day poor outcome by using the logistic algorithm and got a new integrated risk. We then compared the difference between the new integrated risk and the risk assessed by the clinical-radiological model using the Delong test. This study also performed subgroup analysis based on factors related to unmodified factors (sex, age, history of ICCD, renal dysfunction, DAPT history, hematoma location, and GCS score at admission).

## Results

### Baseline information of included patients

This study included 20 SSICH patients with poor outcomes and 20 with good outcomes into the primary cohort after PSM, out of 532 patients (Supplementary Figure 1). Table 1 shows the clinical features of patients in the primary cohort before and after PSM. Of patients in the primary cohort, 24 (60.0%) were male. The median age was 52 years (IQR, 43–62). 31 (77.5%) patients had a history of ICCD. 10 (25.0%) patients took DAPT before hemorrhage. 17 (42.5%) patients received craniotomy or craniotomy plus craniectomy, and 23 (57.5%) received a minimal invasive surgery. Of radiological findings in the primary cohort, the median hematoma volume was 53.8 mL (IQR, 38.7–86.3). 39 (97.5%) hematomas were supratentorial and 1 (2.5%) was infratentorial. No difference was found between patients with poor outcomes and good outcomes in the primary cohort (all *p* > 0.05).

**Table 1:** Baseline information of SSICH patients in the primary cohort before and after PSM.

Features	Pre-PSM			Post-PSM		
	Good outcome *N* = 124	Poor outcome *N* = 20	*p*-value	Good outcome *N* = 20	Poor outcome *N* = 20	*P*-value
Age, m (IQR), y	53 (39–61)	55 (43–62)	0.423	48 (41–61)	55 (43–62)	0.495
Male, no. (%)	104 (83.9%)	7 (35.0%)	0.899	17 (85.0%)	7 (35.0%)	1.000
Comorbidities, no. (%)						
Diabetes mellitus	10 (8.1%)	1 (5.0%)	0.633	1 (5.0%)	1 (5.0%)	0.799
Dyslipidemia	5 (4.0%)	0 (0.0%)	0.362	0 (0.0%)	0 (0.0%)	1.000
History of ICCD	38 (30.6%)	16 (80.0%)	<0.001^┼^	15 (75.0%)	16 (80.0%)	0.799
History of intracerebral hemorrhage	4 (3.2%)	0 (0.0%)	0.417	1 (5.0%)	0 (0.0%)	0.799
Renal dysfunction	33 (26.6%)	6 (30.0%)	0.753	4 (20.0%)	6 (30.0%)	0.602
Current-or-ever smoker, no. (%)	41 (33.1%)	7 (35.0%)	0.865	8 (40.0%)	7 (35.0%)	0.799
Regular drinkers, no. (%)	29 (23.4%)	5 (25.0%)	0.449	7 (35.0%)	5 (25.0%)	0.947
DAPT history, no. (%)	13 (10.5%)	6 (30.0%)	0.017^┼^	4 (20.0%)	6 (30.0%)	0.602
Hematoma location, no. (%)			0.150			0.799
Supratentorial	102 (82.3%)	19 (95.0%)		20 (100.0%)	19 (95.0%)	
Infratentorial	22 (17.7%)	1 (5.0%)		0 (0.0%)	1 (5.0%)	
Hematoma volume, m (IQR), ml	42.2 (32.9–59.9)	77.0 (34.8–104.3)	0.027^┼^	52.3 (41.0–58.2)	77.2 (34.8–104.3)	0.445
IVH, no. (%)	49 (39.5%)	11 (55.0%)	0.194	12 (60.0%)	11 (55.0%)	0.799
Coagulation disorder, no. (%)	0 (0.0%)	0 (0.0%)	1.000	0 (0.0%)	0 (0.0%)	1.000
GCS score at admission, m (IQR)	11 (8–12)	10 (8–12)	0.481	10 (9–11)	10 (8–12)	0.883
Surgical methods, no. (%)			0.762			0.904
Craniotomy/craniotomy + craniectomy	47 (37.9%)	10 (50.0%)		7 (35.0%)	10 (50.0%)	
Minimal invasive surgery	77 (62.1%)	10 (50.0%)		13 (65.0%)	10 (50.0%)	

^┼, the difference is significant.^

SSICH, severe spontaneous intracerebral hemorrhage; PSM, propensity score matching; ICCD, ischemic cerebrovascular or cardiovascular disease; DAPT, dual antiplatelet therapy; IVH, intraventricular hemorrhage GCS, Glasgow coma score.

**Figure 1:**
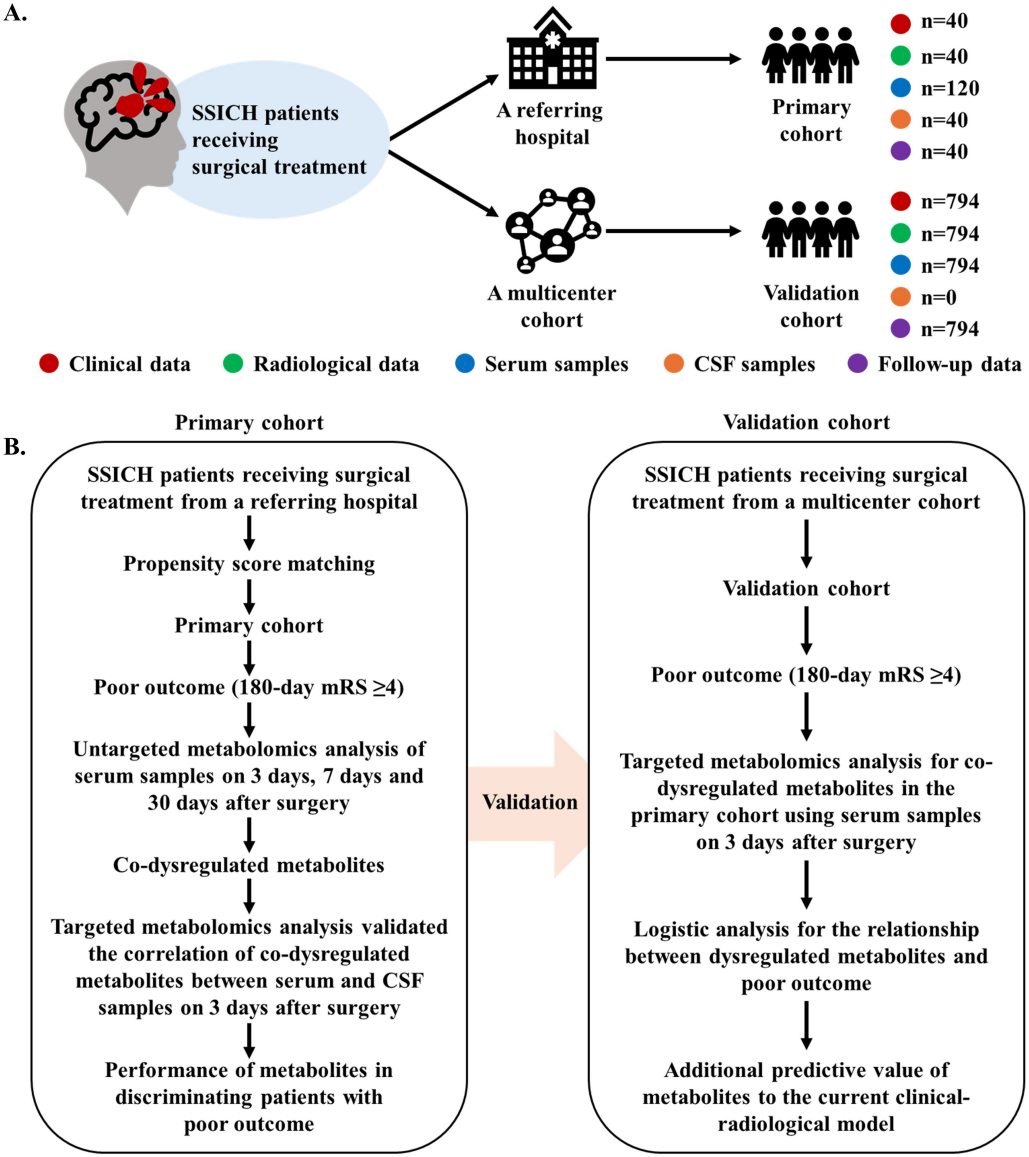
Diagram of study design. **(A)** The summary of data generation in this current study. **(B)** The flowchart of analysis to investigate the metabolism features of SSICH patients with poor outcomes after surgery. This study prospectively included SSICH patients receiving surgery from a referring hospital and formed the primary cohort using propensity score matching. The dynamic change in metabolism features of SSICH patients after surgery and the correlation of dysregulated metabolites between serum and CSF samples were investigated. This study subsequently validated whether dysregulated metabolites is associated with the poor outcomes of SSICH patients after surgery based on a population-based analysis. SSICH, severe spontaneous intracerebral hemorrhage; CSF, cerebrospinal fluid; mRS, modified Rankin scale.

The validation cohort included 794 SSICH patients receiving surgical treatment, out of 1,416 patients (Supplementary Figure 2). 180-day poor outcome was found in 147 (18.5%) patients. Table 2 shows the clinical features of included patients in the validation cohort. Of them, 608 (76.6%) patients were male, and the median age was 54 years (IQR, 44–62). 396 (49.9%) patients had a history of ICCD and 123 (15.5%) had renal dysfunction on admission. 362 (45.6%) patients received craniotomy or craniotomy plus craniectomy, and 432 (54.4%) received a minimal invasive surgery. The median hematoma volume was 55.1 mL (IQR, 36.0–72.9). 710 (89.4%) hematomas were supratentorial and 84 (10.6%) was infratentorial. A significant difference was found in the history of ICCD (*p* = 0.007), renal dysfunction (*p* = 0.038), DAPT history (*p* < 0.001), hematoma volume (*p* < 0.001), IVH (*p* < 0.001), coagulation disorder (*p* = 0.002), GCS at admission (*p* < 0.001) and surgical methods (*p* = 0.002) between patients with poor outcomes and good outcomes in the validation cohort (Figure 1).

**Table 2:** Baseline information of SSICH patients in the validation cohort.

Features	Good outcome *N* = 647	Poor outcome *N* = 147	*P*-value
Age, m (IQR), y	53 (43–62)	55 (46–63)	0.073
Male, no. (%)	497 (76.8%)	111 (75.5%)	0.736
Comorbidities, no. (%)			
Diabetes mellitus	80 (12.4%)	22 (15.0%)	0.395
Dyslipidemia	51 (7.9%)	5 (3.4%)	0.056
History of ICCD	308 (47.6%)	88 (59.9%)	0.007^┼^
History of intracerebral hemorrhage	17 (2.6%)	5 (3.4%)	0.606
Renal dysfunction	92 (14.2%)	31 (21.1%)	0.038^┼^
Current-or-ever smoker, no. (%)	175 (27.0%)	49 (33.3%)	0.127
Regular drinkers, no. (%)	90 (13.9%)	32 (21.8%)	0.087
DAPT history, no. (%)	15 (2.3%)	16 (10.9%)	<0.001^┼^
Hematoma location, no. (%)			0.667
Supratentorial	580 (89.6%)	130 (88.4%)	
Infratentorial	67 (10.4%)	17 (11.6%)	
Hematoma volume, m (IQR), ml	53.0 (35.0–70.7)	63.2 (49.4–89.8)	<0.001^┼^
IVH, no. (%)	322 (49.8%)	110 (74.8%)	<0.001^┼^
Coagulation disorder, no. (%)	37 (5.7%)	17 (11.6%)	0.002^┼^
GCS score at admission, m (IQR)	9 (6–12)	6 (3–11)	<0.001^┼^
Surgical methods, no. (%)			0.002^┼^
Craniotomy/craniotomy + craniectomy	278 (43.0%)	84 (57.1%)	
Minimal invasive surgery	369 (57.0%)	63 (42.9%)	

^┼, the difference is significant.^

SSICH, severe spontaneous intracerebral hemorrhage; ICCD, ischemic cerebrovascular or cardiovascular disease; DAPT, dual antiplatelet therapy; IVH, intraventricular hemorrhage; GCS, Glasgow coma score.

### Dynamic change in metabolism features after surgery for SSICH

**Figure 2:**
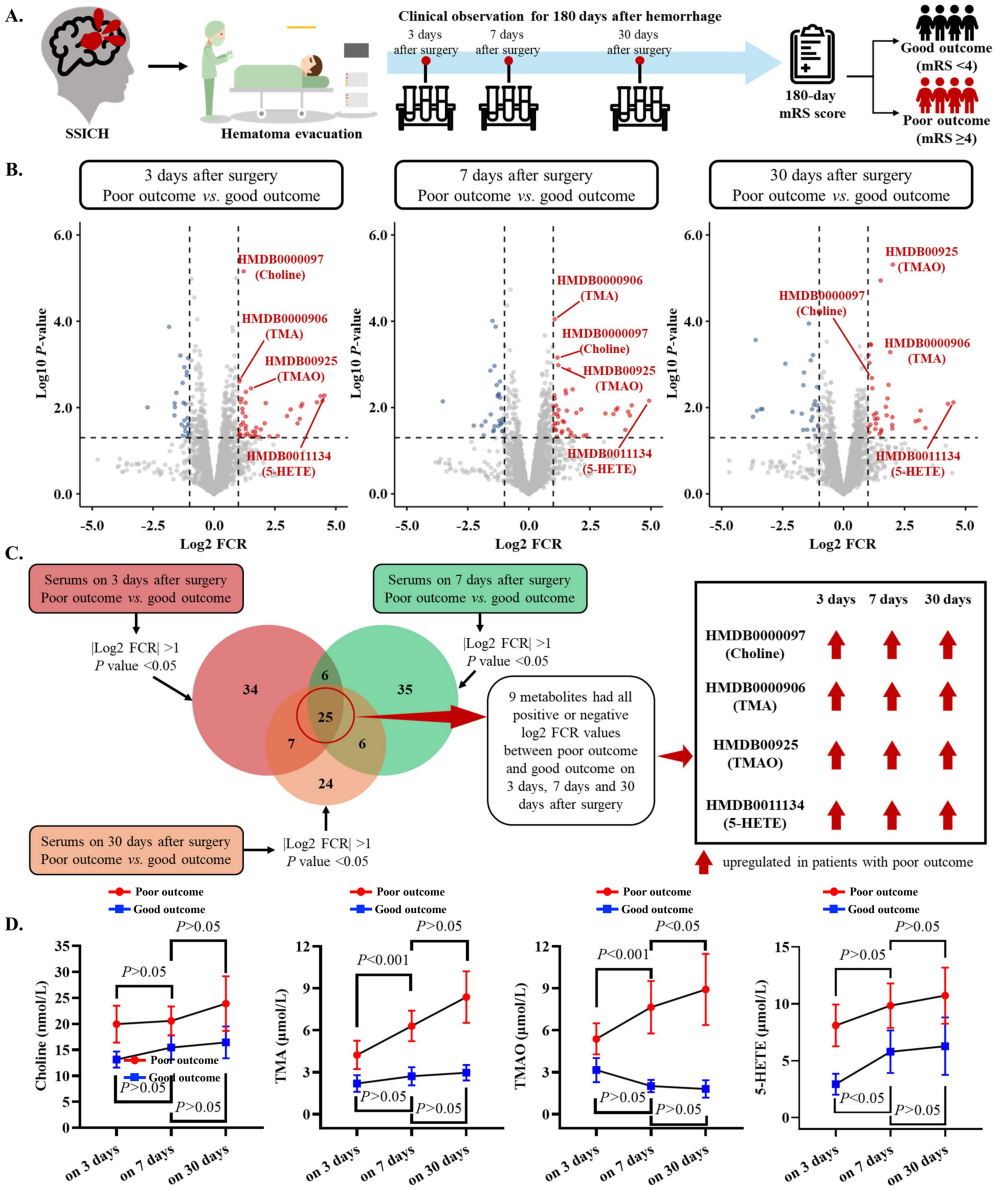
Dynamic change in metabolism features of SSICH patients after surgery based on the primary cohort. **(A)** The diagram of the study aims to investigate the dynamic change in metabolism features of SSICH patients after surgery. **(B)** Volcano plots present the dysregulated metabolites on the 3rd day, 7th day, and 30th day after surgery between SSICH patients with poor outcomes and good outcomes. **(C)** Venn plot presents the co-dysregulated metabolites of 3-day, 7-day, and 30-day metabolism features after surgery between SSICH patients with poor outcomes and good outcomes. This study identified nine metabolites, including Choline, TMA, TMAO, and 5-HETE, with the same trend between ICH patients with poor outcomes and good outcomes on the 3rd day, 7th day, and 30th day after surgery. **(D)** The line plots present the dynamic change of Choline, TMA, TMAO, and 5-HETE in ICH patients with poor outcomes and good outcomes on 3rd day, 7th day, and 30th day after surgery. TMAO had a continuously increasing trend in patients with poor outcome after surgery. SSICH, severe spontaneous intracerebral hemorrhage; mRS, modified Rankin scale; TMA, trimethylamine; TMAO, trimethylamine N-oxide; 5-HETE, 5-Hydroxyeicosatetraenoic acid.

Based on the primary cohort, this study further investigated the dynamic change in metabolic features of SSICH patients postoperatively (Figure 2A). The untargeted metabolomics analysis revealed 72 dysregulated metabolites on the 3rd day after surgery, 72 dysregulated metabolites on the 7th day after surgery, and 62 dysregulated metabolites on the 30th day after surgery, between SSICH patients with poor outcome and good outcome (Figure 2B). Among dysregulated metabolites, they were mainly alcohol, amines and amino acid (Supplementary Figure 3A), and participated in the diet-dependent TMA/TMAO metabolism pathway (Supplementary Figure 3B). Subsequent analysis revealed 25 co-dysregulated metabolites among 3-day, 7-day, and 30-day metabolism features after surgery; 9 metabolites had the same trend, including choline, TMA, TMAO and 5-HETE, between SSICH patients with poor outcome and good outcome on 3rd day, 7th day, and 30th day after surgery (Figure 2C and Supplementary Figures 3C,D). Targeted metabolomics analysis only validated the difference of four metabolites, including choline, TMA, TMAO and 5-HETE, between patients with poor outcomes and good outcomes (Supplementary Table 1). A continuously increasing trend was found in TMA and TMAO of patients with poor outcomes from the 3rd day to the 30th day after surgery, whereas no similar result was found in choline and 5-HETE (Figure 2D).

This study subsequently investigated the correlation of co-dysregulated metabolites (including choline, TMA, TMAO, and 5-HETE) between serum and CSF samples on the 3^rd^ day after surgery (Supplementary Figure 4A). A good correlation was found in choline, TMA, TMAO, and 5-HETE between serum and CSF on the 3rd day after surgery (all *r* > 0.6) (Supplementary Figure 4B). Further analysis also showed that TMAO level on 3rd day after surgery performed best to classify SSICH patients with poor outcomes from good outcomes (Supplementary Figure 5).

### High serum TMAO level is related to poor outcome in SSICH patients

**Figure 3:**
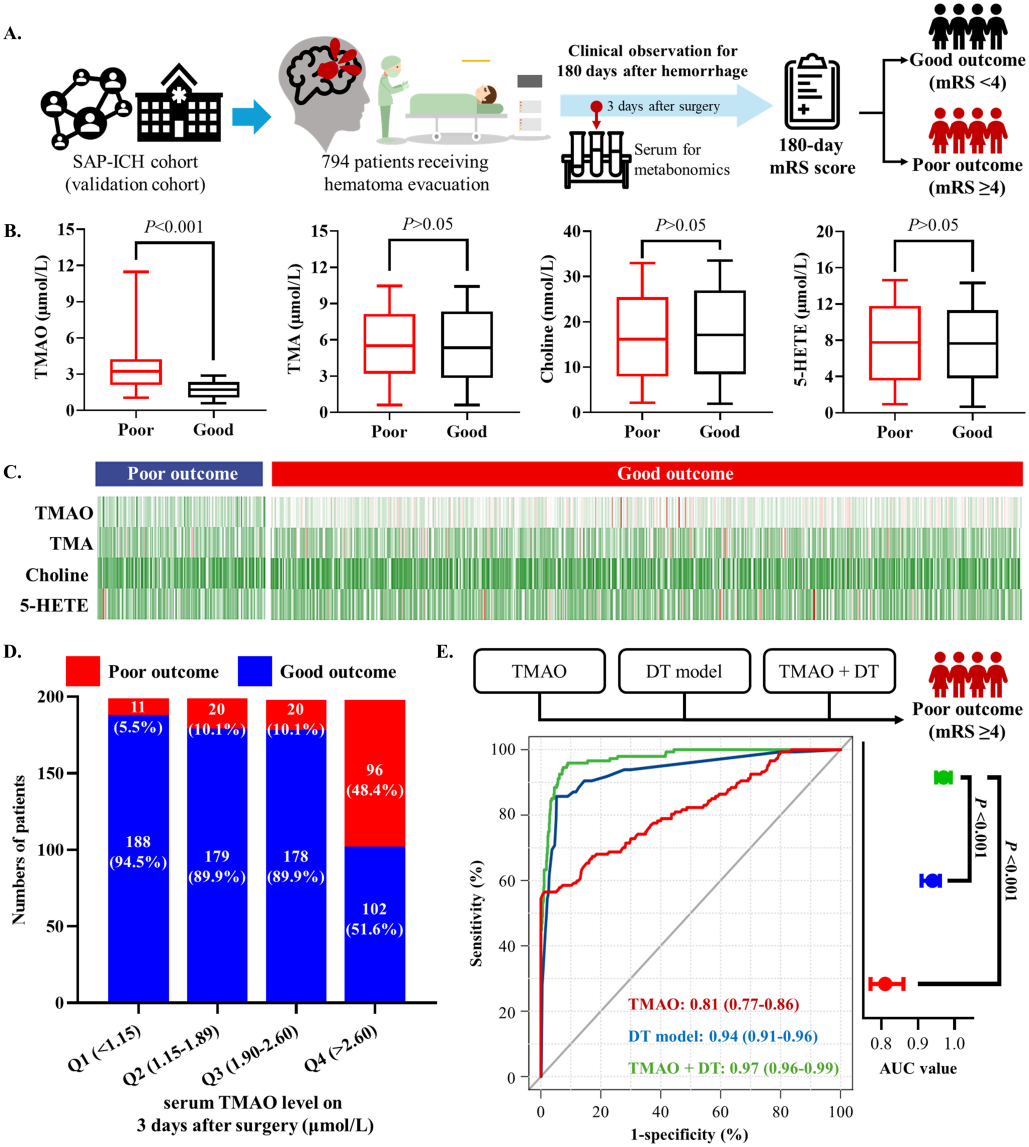
High serum TMAO level is related to poor outcomes in SSICH patients after surgery based on the validation cohort. **(A)** The diagram of study to investigate whether dysregulated metabolites are related to poor outcome of SSICH patients after surgery in validation cohort. **(B)** Box plots present the dysregulated metabolites on 3rd day after surgery between SSICH patients with poor outcomes and good outcomes in the validation cohort. **(C)** Feature map presents the co-dysregulated metabolites of 3-day metabolism features between SSICH patients with poor outcomes and good outcomes after surgery in the validation cohort. **(D)** The bar chart presents the incidence of poor outcome in different TMAO levels. TMAO had a continuously increasing trend in patients with poor outcomes after surgery. **(E)** The ROC curve demonstrates that combining TMAO and the DT model yielded a higher AUC in predicting poor outcomes in SSICH patients. SSICH, severe spontaneous intracerebral hemorrhage; mRS, modified Rankin scale; TMA, trimethylamine; TMAO, trimethylamine N-oxide; 5-HETE, 5-Hydroxyeicosatetraenoic acid; ROC, receiver operate curve; AUC, area under curve.

Based on the validation cohort, this study further investigated whether dysregulated metabolites are related to poor outcomes of SSICH patients after surgery (Figure 3A). The targeted metabolomics analysis found significant difference in TMAO on the 3rd day after surgery between patients with poor outcomes and good outcomes (*p* < 0.001); and, no significant difference was found in choline, TMA and 5-HETE between patients with poor outcomes and good outcomes (all *p* > 0.05) (Figure 3B). The feature map of choline, TMA, TMAO, and 5-HETE level on the 3rd day after surgery in patients with poor outcomes and good outcomes was shown in Figure 3C.

Logistic analysis revealed TMAO level on the 3rd day after surgery as the risk factor of 180-day poor outcome (OR, 4.7; 95%CI, 3.6–6.2; *p* < 0.001; Table 3). All patients in the validation cohort were categorized as four groups according to the interquartile range of TMAO level on the 3rd day after surgery (as Q1–Q4). The patients with TMAO Q4 (>2.60 μmol/L) had the highest percentage of poor outcomes, followed by patients with other TMAO levels (Figure 3D). Univariate logistic analysis showed TMAO on the 3rd day after surgery >2.60 μmol/L as a risk factor of poor outcomes (OR, 16.1; 95%CI, 8.2–31.4; *p* < 0.001; Table 3). The result was consistent in TMAO >2.60 μmol/L for the poor outcomes after surgery (adjusted OR, 15.8; 95%CI, 8.0–31.3; *p* < 0.001), being adjusted by the history of ICCD, renal dysfunction, DAPT history, hematoma volume and GCS score at admission (Table 3).

**Table 3:** Logistic regression analysis for poor outcome in the validation cohort.

TMAO level on the 3rd day after surgery	Raw logistic model		Adjusted logistic model	
	ORs (95%CI)	*P*-value	ORs (95%CI)	*P*-value
Continuous TMAO level	4.7 (3.6–6.2)	<0.001	4.4 (3.3–5.8)	<0.001
Interquartile range of TMAO level				
Q1 (<1.15 μmol/L)	Reference		Reference	
Q2 (1.15–1.89 μmol/L)	1.9 (0.9–4.1)	0.097	2.1 (0.9–4.5)	0.064
Q3 (1.90–2.60 μmol/L)	1.9 (0.9–4.1)	0.094	2.0 (0.9–4.3)	0.082
Q4 (>2.60 μmol/L)	16.1 (8.2–31.4)	<0.001	15.8 (8.0–31.3)	<0.001

The logistic model was adjusted by history of ICCD, renal dysfunction, DAPT history, hematoma volume and GCS score at admission.

TMAO, trimethylamine N-oxide; DAPT, dual antiplatelet therapy; ICCD, ischemic cerebrovascular or cardiovascular disease; GCS, Glasgow coma score; OR, odds ratio.

Serum TMAO level on the 3rd day after surgery performed well to predict the risk of 180-day poor outcome of SSICH patients (AUC, 0.81; 95%CI, 0.77–0.86; *p* < 0.001), and could provide additional predictive value to our previous clinical-radiological DT model (AUC from 0.94 to 0.97, *p* < 0.001; Figure 3E).

Subgroup analysis on unmodified factors related to the poor outcome of SSICH patients after surgery (including sex, age, history of ICCD, renal dysfunction, DAPT history, hematoma location and GCS score at admission) also confirmed that patients with increased TMAO on the 3rd day after surgery had a high risk of suffering from the poor outcome (Supplementary Table 2).

## Discussion

Investigation of metabolic characteristics of SSICH patients after surgical treatment could reveal the biomarkers of poor outcomes. In this study, it was found that choline, TMA, TMAO, and 5-HETE were metabolites associated with 180-day poor outcomes in SSICH patients via analyzing the postoperative dynamic change of SSICH patients. Based on a population-based cohort, TMAO as the metabolite of 180-day poor outcomes was further validated. Serum TMAO level on the 3^rd^ day after surgery performed well to predict the risk of 180-day poor outcome in SSICH patients and could also provide additional predictive value to our previous clinical-radiological DT model (AUC from 0.94 to 0.97, *p* < 0.001). The current study revealed that TMAO could serve as the early-warning biomarker for 180-day poor outcomes in SSICH patients.

Metabolic conditions are usually unstable after surgery. Identification of metabolite with stable difference between patients with poor outcomes and good outcomes is key to reveal the biomarkers for 180-day poor outcomes. In this study, the metabolic characteristics of SSICH after surgery were dynamically monitored, and it was found that altered metabolites mainly participated in TMA/TMAO metabolism pathway. Subsequent analysis showed 4 metabolites, including TMAO, with stable differences between SSICH patients with poor outcomes and good outcomes on the 3rd day, 7th day, and 30th day after surgery. A good correlation of the expression level of these 4 metabolites between serum and CSF was also confirmed. Based on these above facts, we could effectively discover the biomarkers of 180-day poor outcomes in SSICH patients after surgery.

TMAO, a metabolite derived from choline, has been strongly correlated with adverse events and poor prognosis in numerous studies (17–22). Evidence from a multi-center cohort observed a strong association between elevated TMAO and the incidence of major adverse cardiac events (19). Moreover, Zhai et al. (20) revealed that high levels of TMAO are associated with poor outcomes in patients with intracerebral hemorrhage. Several *in vivo* and *in vitro* studies suggested that TMAO could induce neuroinflammation and endothelial dysfunction, which subsequently intensified inflammation (9, 23). Recent studies suggest elevated TMAO levels causes upregulation of ROS, hydrogen peroxide, lipid peroxidation etc. resulting in oxidative stress, therefore deteriorating endothelial function (24, 25). Furthermore, previous studies have shown that elevated TMAO levels increase the risk of atherothrombosis and atherosclerotic plaque instability (22, 26). In our research, a strong association between elevated TMAO levels and poor postoperative outcomes in patients with SSICH was identified. The mechanisms by which elevated TMAO levels contribute to poor patient prognosis may include several factors. As a pro-inflammatory factor, increased levels of TMAO after surgery suggest an intensified inflammatory response in patients, which may lead to a worse prognosis (27, 28). Furthermore, patients with SSICH frequently have atherosclerosis, and elevated TMAO levels heighten the risk of plaque rupture, potentially leading to adverse cardiovascular or cerebrovascular events such as stroke and acute myocardial infarction, which significantly and negatively affect patient prognosis (29–31).

This study demonstrated that TMAO could provide additional predictive value to the clinical-radiological model for 180-day poor outcomes in SSICH patients after surgery. The previous model, which relied on clinical and radiological features of SSICH patients, which did not consider patients’ internal environment. As mentioned above, TMAO could serve as an inflammation-related biomarker and could reflect the brain injury level of SSICH patients after surgery. The clinical and radiological characteristics are indicative of the severity of the baseline condition of SSICH patients, while metabolic biomarkers provide insights into the dynamic changes in the internal environment and brain injury following SSICH surgery. Consequently, combining clinical, radiological, and metabolic features related to SSICH provides improved predictive capability for patient prognosis. Elevated TMAO levels could serve as an early warning signal for patients at greater risk of developing poor outcomes, such as post-surgical complications or extended recovery times. Patients with higher TMAO levels might benefit from more aggressive anti-inflammatory treatments or closer monitoring for complications related to brain injury. This approach could help clinicians better anticipate patient outcomes, tailor treatment plans, and allocate resources more effectively, ultimately leading to more personalized and precise care for SSICH patients.

This study has several limitations. First, the cohort was exclusively Chinese, which might limit the generalizability of the results to other populations. Second, the study was susceptible to multi-center bias due to the involvement of multiple research centers, potentially introducing variations in study protocols and patient characteristics. Third, the specific impact of different surgical procedures on patient outcomes was not examined in detail, as our primary goal was to investigate biomarkers for postoperative poor outcomes. Finally, there were potential confounding factors that were not incorporated into the analysis, which could influence the study outcomes. Factors such as diet, gut microbiota, and medications (e.g., antibiotics) have been shown to affect serum TMAO levels, and their influence could not be controlled for in this study. For example, dietary intake of certain nutrients, such as choline and L-carnitine, could impact the production of TMAO through gut microbial metabolism. Antibiotic use could also alter the gut microbiome, potentially influencing TMAO levels and its associated outcomes.

## Conclusion

This study on metabolomics analysis demonstrated that increasing serum TMAO level was associated with the 180-day poor outcome of SSICH patients receiving surgical treatment, which could serve as a biomarker for poor outcomes.

## Data Availability

The raw data supporting the conclusions of this article will be made available by the authors, without undue reservation.

## Footnotes

^1^ http://www.metware.cn/

^2^ https://www.metaboanalyst.ca/
