# Supplementary material for: Metabolomic analysis reveals trimethylamine N-oxide as a biomarker for poor outcome of severe spontaneous intracerebral hemorrhage patients receiving surgical treatment
Source: Front Neurol. 2025 Apr 14;16:1551239. doi: 10.3389/fneur.2025.1551239 (PMC12034627; doi:10.3389/fneur.2025.1551239)
Supplement: Supplementary file 1 [file Data_Sheet_1.DOCX]

**Supplemental materials fo**r “*Metabonomic analysis reveals trimethylamine N-oxide as a biomarker for poor outcome of severe spontaneous intracerebral hemorrhage patients*”

**Supplemental tables**

**Supplemental table 1. Targeted metabonomic analysis of dysregulated metabolite based on the primary cohort.**

| Metabolite index | Good outcome  N=20 | Poor outcome  N=20 | *P* value |
| --- | --- | --- | --- |
| **On 3^rd^ day** |  |  |  |
| TMAO, m (IQR), μmol/L | 2.6 (1.7-5.2) | 5.5 (3.2-6.7) | <0.001 |
| TMA, m (IQR), μmol/L | 1.9 (1.1-3.0) | 3.9 (2.7-6.2) | <0.001 |
| 5-HETE, m (IQR), μmol/L | 2.3 (1.3-4.7) | 7.6 (5.1-11.9) | <0.001 |
| Choline, m (IQR), nmol/L | 13.4 (10.7-15.7) | 20.3 (13.3-26.9) | <0.001 |
| Lactic acid, m (IQR) | 77.6 (41.4-164.9) | 107.4 (52.3-153.9) | 0.314 |
| Lupulone, m (IQR) | 160.5 (49.7-427.4) | 351.3 (95.6-902.8) | 0.114 |
| Etiocholanolone glucuronide, m (IQR) | 880.2 (673.9-1398.9) | 668.9 (402.5-992.1) | 0.080 |
| Ochratoxin A, m (IQR) | 27.1 (8.7-129.1) | 90.7 (33.6-158.8) | 0.072 |
| {[5-({4,5-dihydroxy-2-[4-(7-hydroxy-4-oxo-3,4-dihydro-2H-1-benzopyran-2-yl)phenoxy]-6-(hydroxymethyl)oxan-3-yl}oxy)-3,4-dihydroxyoxolan-3-yl]methoxy}sulfonic acid, m (IQR) | 2772.1 (1302.1-3769.8) | 3189.4 (2482.7-4388.4) | 0.157 |
| **On 7^th^ day** |  |  |  |
| TMAO, m (IQR), μmol/L | 2.1 (1.3-2.7) | 6.0 (4.8-11.4) | <0.001 |
| TMA, m (IQR), μmol/L | 3.0 (1.6-3.9) | 6.5 (4.6-8.2) | <0.001 |
| 5-HETE, m (IQR), μmol/L | 4.8 (3.0-6.5) | 10.1 (6.5-12.4) | <0.001 |
| Choline, m (IQR), nmol/L | 16.3 (10.7-18.9) | 19.3 (17.6-23.7) | <0.001 |
| Lactic acid, m (IQR) | 70.4 (36.9-168.0) | 125.5 (73.4-197.5) | 0.174 |
| Lupulone, m (IQR) | 16.0 (60.6-321.0) | 274.0 (99.4-852.5) | 0.091 |
| Etiocholanolone glucuronide, m (IQR) | 811.0 (671.0-1285.0) | 706.0 (361.0-938.0) | 0.221 |
| Ochratoxin A, m (IQR) | 37.3 (7.2-138.0) | 106 (30.3-208.0) | 0.063 |
| {[5-({4,5-dihydroxy-2-[4-(7-hydroxy-4-oxo-3,4-dihydro-2H-1-benzopyran-2-yl)phenoxy]-6-(hydroxymethyl)oxan-3-yl}oxy)-3,4-dihydroxyoxolan-3-yl]methoxy}sulfonic acid, m (IQR) | 2445.3 (1239.6-3673.6) | 3565.5 (2162.3-4346.2) | 0.096 |
| **On 30^th^ day** |  |  |  |
| TMAO, m (IQR), μmol/L | 1.9 (0.5-2.9) | 7.6 (5.1-10.0) | <0.001 |
| TMA, m (IQR), μmol/L | 2.9 (2.0-4.1) | 8.9 (5.4-11.2) | <0.001 |
| 5-HETE, m (IQR), μmol/L | 5.2 (2.6-8.1) | 11.0 (6.8-14.6) | <0.001 |
| Choline, m (IQR), nmol/L | 14.0 (12.3-19.7) | 22.9 (14.1-34.8) | <0.001 |
| Lactic acid, m (IQR) | 73.2 (40.7-155.5) | 97.1 (47.7-163.9) | 0.583 |
| Lupulone, m (IQR) | 142.9 (52.4-295.0) | 380.8 (112.7-782.9) | 0.072 |
| Etiocholanolone glucuronide, m (IQR) | 793.1 (701.9-1264.5) | 623.9 (452.4-913.6) | 0.043 |
| Ochratoxin A, m (IQR) | 21.4 (8.2-97.4) | 66.6 (23.7-168.0) | 0.049 |
| {[5-({4,5-dihydroxy-2-[4-(7-hydroxy-4-oxo-3,4-dihydro-2H-1-benzopyran-2-yl)phenoxy]-6-(hydroxymethyl)oxan-3-yl}oxy)-3,4-dihydroxyoxolan-3-yl]methoxy}sulfonic acid, m (IQR) | 2689.1 (1129.1-3505.5) | 3449.3 (2225.8-4220.0) | 0.081 |

TMAO, TMA, 5-HETE and choline in serum were detected by using the multiple reaction monitoring based on the standard samples. Other metabolites in serum were detected by using the multiple reaction monitoring based on the signal intensity.

TMA, trimethylamine; TMAO, trimethylamine N-oxide; 5-HETE, 5-Hydroxyeicosatetraenoic acid.

**Supplemental table 2. Serum TMAO level on 3^rd^ day after surgery and risk of 180-day poor outcome within the validation cohort, stratified by various factors related to poor outcome.**

| Groups | Total  patients | Patients with  Event (*no.*) | Incidence  (%) | Odds ratio  (95%CI) | *P* value |
| --- | --- | --- | --- | --- | --- |
| All | 794 | 147 | 18.5 | 4.7 (3.6-6.2) | <0.001 |
| Male | 608 | 111 | 18.3 | 4.7 (3.4-6.4) | <0.001 |
| Female | 186 | 36 | 19.4 | 4.9 (2.8-8.7) | <0.001 |
| Age |  |  |  |  |  |
| > 70 years old | 52 | 20 | 38.5 | 3.9 (1.8-8.6) | 0.001 |
| ≤ 70 years old | 742 | 127 | 17.1 | 4.7 (3.5-6.3) | <0.001 |
| History of ICCD |  |  |  |  |  |
| Yes | 396 | 88 | 22.2 | 4.8 (3.4-7.0) | <0.001 |
| No | 398 | 59 | 14.8 | 4.4 (2.9-6.7) | <0.001 |
| Renal dysfunction |  |  |  |  |  |
| Yes | 123 | 31 | 25.2 | 5.2 (2.60-10.3) | 0.001 |
| No | 671 | 116 | 17.3 | 4.8 (3.5-6.4) | <0.001 |
| DAPT history |  |  |  |  |  |
| Yes | 31 | 16 | 51.6 | 3.9 (1.5-10.1) | <0.001 |
| No | 763 | 131 | 17.2 | 4.7 (3.5-6.3) | <0.001 |
| Hematoma location |  |  |  |  |  |
| Supratentorial | 710 | 130 | 18.3 | 6.3 (2.6-14.9) | <0.001 |
| Infratentorial | 84 | 17 | 20.2 | 4.6 (3.4-6.1) | <0.001 |
| GCS score at admission |  |  |  |  |  |
| ≥ 8 | 466 | 59 | 12.7 | 5.9 (3.7-9.4) | <0.001 |
| < 8 | 328 | 88 | 26.8 | 3.9 (2.8-5.4) | <0.001 |
| Surgical methods |  |  |  |  |  |
| Craniotomy/craniotomy + craniectomy | 362 | 84 | 23.2 | 3.9 (2.8-5.6) | <0.001 |
| Minimal invasive surgery | 432 | 63 | 14.6 | 6.2 (4.0-9.7) | <0.001 |

ICCD, ischemic cerebrovascular or cardiovascular diseases; DAPT, dual antiplatelet therapy; GCS, Glasgow coma score; TMAO, trimethylamine N-oxide.

**Supplemental figures**


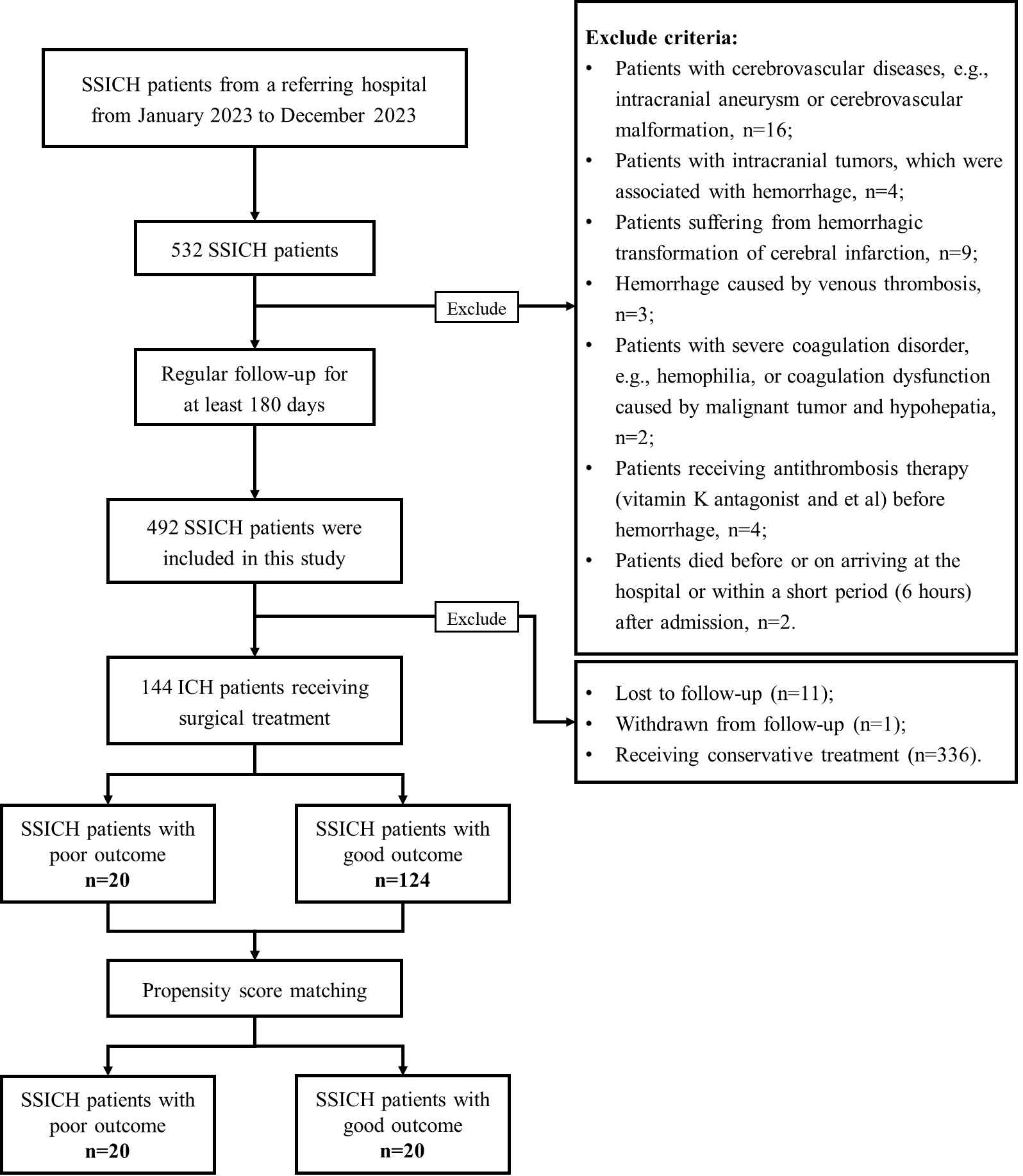


**Supplemental figure 1. Flowchart of patient enrollment in the primary cohort.**


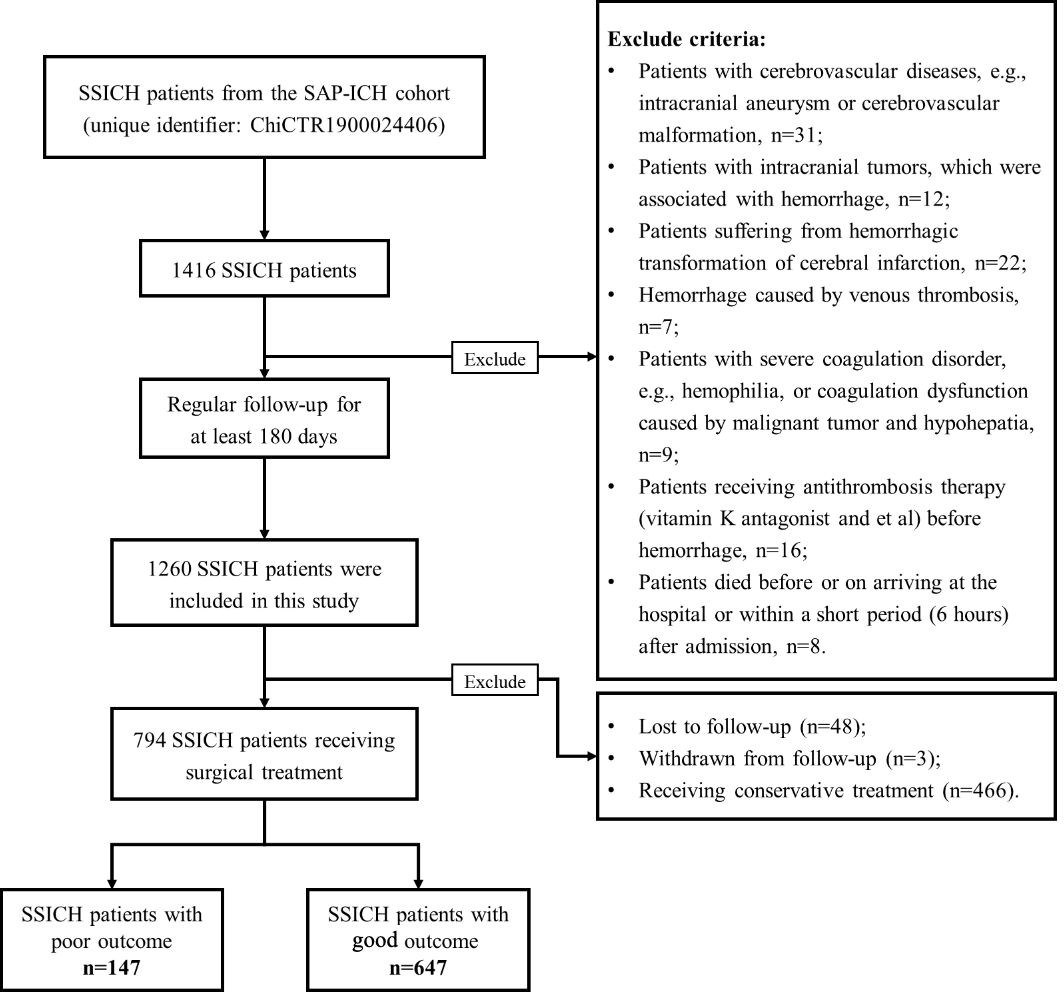


**Supplemental figure 2. Flowchart of patient enrollment in the validation cohort.**


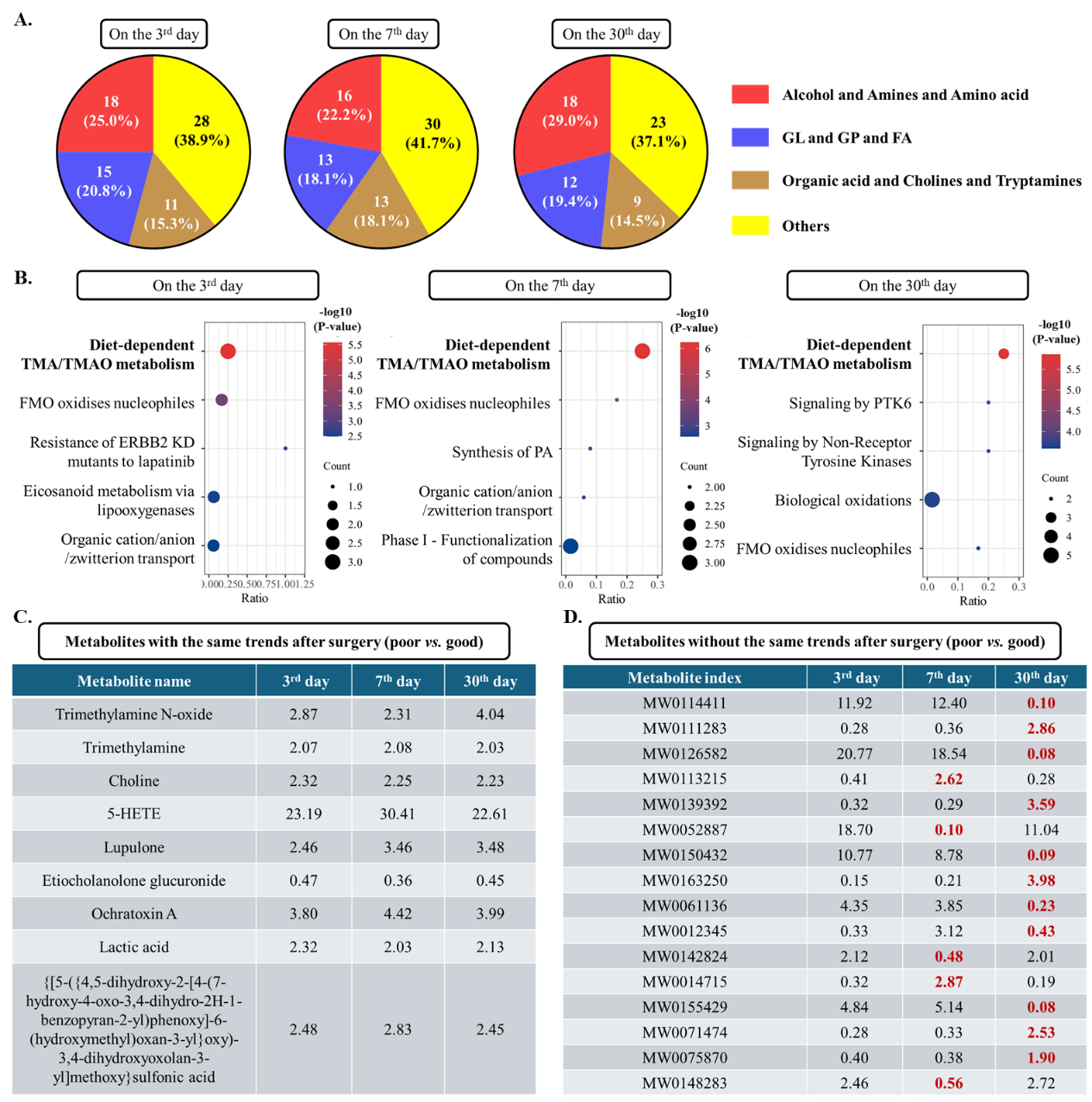


**Supplemental figure 3. Enrichment analysis of dysregulated metabolites in SSICH patients after surgery based on the primary cohort.**

1. The pie plots present the dysregulated metabolites on 3^rd^ day, 7^th^ day and 30^th^ day after surgery between SSICH patients with poor outcome and good outcome.
2. The bubble plots present the pathway enrichment analysis of dysregulated metabolites on 3^rd^ day, 7^th^ day and 30^th^ day after surgery between SSICH patients with poor outcome and good outcome.
3. The table presents 9 metabolites with the same trend between SSICH patients with poor outcome and good outcome on 3^rd^ day, 7^th^ day and 30^th^ day after surgery.
4. The table shows 16 metabolites without the same trend between SSICH patients with poor outcome and good outcome on 3^rd^ day, 7^th^ day and 30^th^ day after surgery.

CSF, cerebrospinal fluid; TMA, trimethylamine; TMAO, trimethylamine N-oxide.


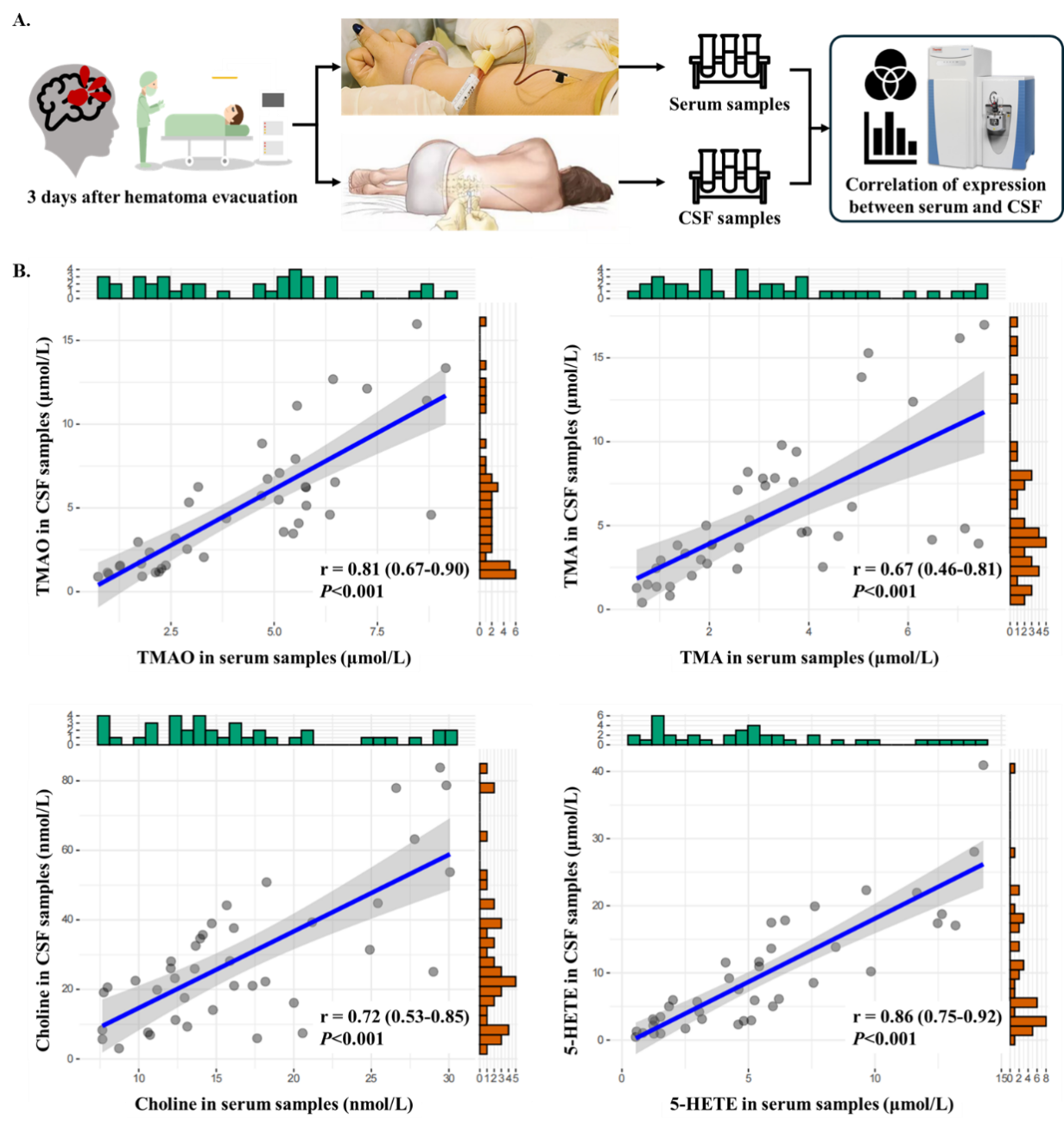


**Supplemental figure 4. The correlation of co-dysregulated metabolites between serum and CSF samples on 3^rd^ day after surgery based on the primary cohort.**

1. The diagram presents the flowchart of study to investigate the correlation of co-dysregulated metabolites between serum and CSF samples.
2. The scatter plots present the correlation of co-dysregulated metabolites between serum and CSF samples. A good correlation was found in TMAO, TMA, choline and 5-HETE between serum and CSF samples, with correlation coefficient >0.6.

CSF, cerebrospinal fluid; TMA, trimethylamine; TMAO, trimethylamine N-oxide.


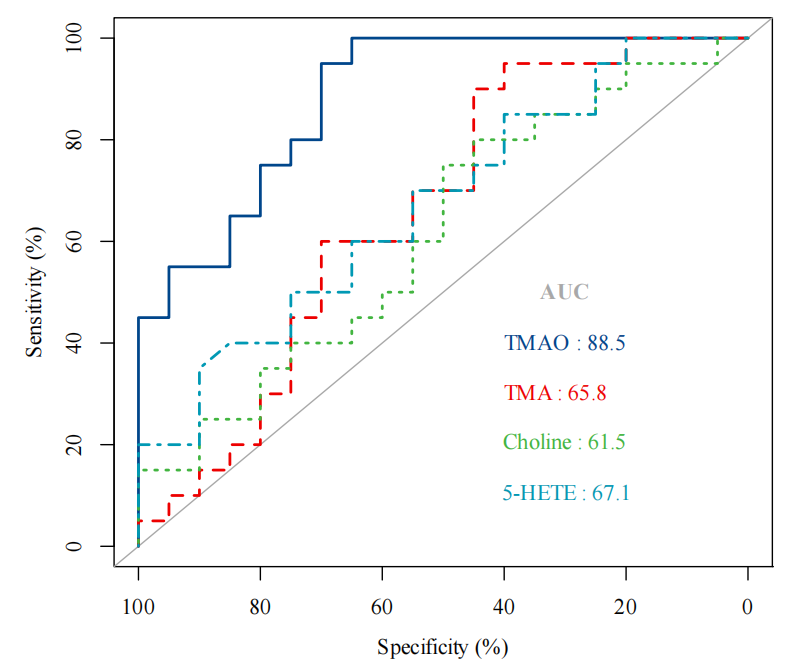


**Supplemental figure 5. The performance of four metabolites on the 3^rd^ day after surgery for classifying SSICH patients with poor outcome and good outcome after surgery.**

The level of TMAO, TMA, choline and 5-HETE were detected by using the targeted metabonomic analysis. The TMAO level on the 3^rd^ day after surgery performed best to classify SSICH patients with poor outcome and good outcome after surgery.

SSICH, severe spontaneous intracerebral hemorrhage; TMA, trimethylamine; TMAO, trimethylamine N-oxide; 5-HETE, 5-Hydroxyeicosatetraenoic acid.
